# Contributions of Ccr4 and Gcn2 to the Translational Response of *C. neoformans* to Host-Relevant Stressors and Integrated Stress Response Induction

**DOI:** 10.1128/mbio.00196-23

**Published:** 2023-04-05

**Authors:** Corey M. Knowles, David Goich, Amanda L. M. Bloom, Murat C. Kalem, John C. Panepinto

**Affiliations:** a Department of Microbiology and Immunology, Witebsky Center for Microbial Pathogenesis and Immunology, Jacobs School of Medicine and Biomedical Sciences, University at Buffalo, Buffalo, New York, USA; University of British Columbia

**Keywords:** integrated stress response, ribosome collision, stress adaptation, translational control

## Abstract

In response to the host environment, the human pathogen Cryptococcus neoformans must rapidly reprogram its translatome from one which promotes growth to one which is responsive to host stress. In this study, we investigate the two events which comprise translatome reprogramming: the removal of abundant, pro-growth mRNAs from the translating pool, and the regulated entry of stress-responsive mRNAs into the translating pool. Removal of pro-growth mRNAs from the translating pool is controlled primarily by two regulatory mechanisms, repression of translation initiation via Gcn2, and decay mediated by Ccr4. We determined that translatome reprogramming in response to oxidative stress requires both Gcn2 and Ccr4, whereas the response to temperature requires only Ccr4. Additionally, we assessed ribosome collision in response to host-relevant stress and found that collided ribosomes accumulated during temperature stress but not during oxidative stress. The phosphorylation of eIF2α that occurred as a result of translational stress led us to investigate the induction of the integrated stress response (ISR). We found that eIF2α phosphorylation varied in response to the type and magnitude of stress, yet all tested conditions induced translation of the ISR transcription factor Gcn4. However, Gcn4 translation did not necessarily result in canonical Gcn4-dependent transcription. Finally, we define the ISR regulon in response to oxidative stress. In conclusion, this study begins to reveal the translational regulation in response to host-relevant stressors in an environmental fungus which is capable of adapting to the environment inside the human host.

## INTRODUCTION

Cryptococcus neoformans is a human-pathogenic fungus which, if not cleared from the lung, can cause cryptococcal meningoencephalitis, which kills nearly 180,000 people annually worldwide (1). Upon inhalation by the human host, C. neoformans is suddenly subjected to a multitude of stressors, including but not limited to the mammalian core temperature of 37°C and oxidative stress produced by macrophages in the lungs. To adapt to and survive the stress from such an extreme environmental transition, C. neoformans must reprogram its translatome from one which promotes cell growth to one which promotes the translation of mRNAs coding for effectors of stress mitigation (2–5).

**FIG 1:**
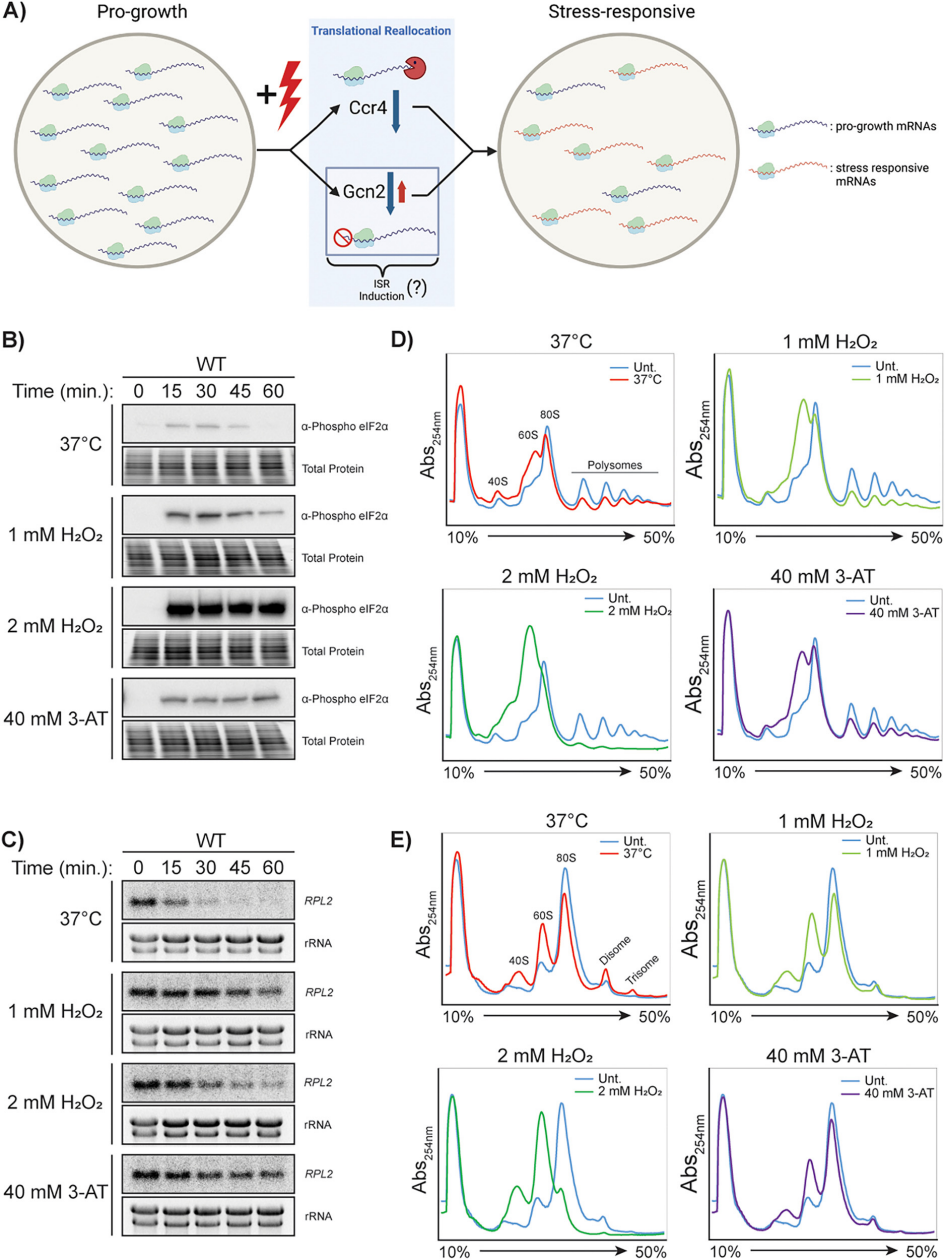
Cryptococcus neoformans represses translation in response to temperature and oxidative stresses. (A) A model for stress responsive translatome reprogramming in which Gcn2 and Ccr4 control the entry and exit of mRNAs from the translating pool, respectively. Western blots for phosphorylated eIF2α (B) and northern blots for the *RPL2* transcript (C) in C. neoformans in response to a shift to 37°C, 1 mM H_2_O_2_, 2 mM H_2_O_2_, and 40 mM 3-AT (3-amino-1,2,4-triazole). (D) Polysome profiling of C. neoformans in response to a shift to 37°C, 1 mM H_2_O_2_, 2 mM H_2_O_2_, and 40 mM 3-AT. (E) Polysome profiling analysis of samples from panel C after treatment with RNase I.

Translatome reprogramming is primarily comprised of two molecular processes: first, the removal of abundant mRNAs that are efficiently translated from the translating pool through mRNA decay, and second, the regulation of translation initiation of mRNAs entering the translating pool. Ribosomal protein (RP) mRNAs and other ribosome biogenesis factors have been shown to comprise the majority of repressed mRNAs and are coordinately regulated during temperature stress and osmotic stress in Saccharomyces cerevisiae and during temperature stress in C. neoformans (3, 6–8). The removal of these abundant mRNAs from the translating pool frees translational resources to allow for the rapid translation of stress-responsive mRNAs (3, 6, 7) (Fig. 1A). Both of these molecular mechanisms operate simultaneously during the stress response, although the contribution from each of these in response to host-relevant stressors is unknown in C. neoformans.

The removal of mRNAs from the translating pool occurs primarily through coordinated mRNA decay, for which the initial and rate-limiting step is deadenylation, performed by Ccr4 in C. neoformans (9). Loss of Ccr4 in C. neoformans severely attenuates virulence and results in broad stress-sensitivity, suggesting a role for deadenylation-dependent mRNA decay beyond that of temperature stress (3, 4, 10).

The regulation of translation initiation occurs primarily via the alpha subunit of eIF2, which is phosphorylated by Gcn2, the sole eIF2α kinase in C. neoformans (5). This results in a reduction in the availability of the ternary ribonucleoprotein complex, which consists of an initiator methionyl-tRNA and eIF2-GTP, to bind to the 40S ribosome to form the 43S pre-initiation complex (11) and results in the global repression of translation initiation (5). When both translation initiation and programmed mRNA decay occur simultaneously, the translatome is reprogrammed for the production of stress-responsive effectors (7). We have previously reported a role of Gcn2 in the response to oxidative stress and during utilization of methionine as a sole nitrogen source in C. neoformans (5, 12). With these two pathways functioning simultaneously to reprogram the translatome, there remains a gap in knowledge about their relative contributions to temperature stress and oxidative stress responses and whether one mechanism can compensate for the other.

Translatome reprogramming is required for the rapid production of stress-response effectors which re-tool the cell to respond to the stress and mitigate damage from the stressor. In the face of mild to moderate stress, these responses are transient, with the goal of restoring normal, unstressed growth. One example of a stress response pathway that requires translatome reprogramming is the integrated stress response (ISR) which is conserved across eukaryotes. In addition to global repression of translation initiation, eIF2α phosphorylation simultaneously results in the induction of the integrated stress response (ISR) by promoting the translation of the stress-response transcription factor Gcn4 (13, 14). In S. cerevisiae, eIF2α phosphorylation results in the 43S scanning pre-initiation complex to bypass upstream open reading frames (uORFs) of the mRNA encoding Gcn4, which typically repress its translation (15). Newly translated Gcn4 then translocates to the nucleus, activating the transcription of stress-responsive mRNAs (16). Gcn4 target genes in S. cerevisiae, which comprise the ISR regulon, are then introduced into the translating pool, facilitating stress adaptation and repression of ribosome biogenesis (17–19). Both the global translation repression and the transcription of stress-responsive mRNAs through the induction of the ISR occur simultaneously as a result of Gcn2 activation, allowing for an efficient cellular response to and mitigation of the stressor. At this time, the ISR has not been defined in C. neoformans, and critically, it is not known whether host-relevant stressors trigger ISR induction.

A growing body of evidence has begun to uncover how environmental stress is sensed by cells. Osmotic stress, heat shock, and stress from translation inhibitors, RNA-damaging chemicals, tRNA availability, and mRNA defects have all been shown to result in stalled or collided ribosomes (20–27). Stalled or collided ribosomes serve as a trigger and signaling platform for various downstream events, including Gcn2 activation (24, 28–31) and subsequent ISR induction, ribosome quality control, nascent polypeptide degradation, and mRNA surveillance and decay (26, 32–42). However, ribosome collisions and their downstream consequences in fungal pathogens have yet to be described. In this study, we sought to determine which stressors lead to translational stress-induced ribosome collision and to characterize the contribution from both regulation of mRNA entry and exit of the translating pool upon translatome reprogramming in C. neoformans. We assessed host-relevant stressors and determined which stressors lead to translational stress-induced ribosome collision. In addition to examining translational regulation in response to individual physiologically relevant stressors, we also began to probe responses to compound stress that may mimic the host environment more closely. Finally, we examined how stress-induced translation regulation induces the ISR and define the ISR regulon in C. neoformans.

## RESULTS

### *C. neoformans* exhibits ribosome collision and translational repression in response to temperature and oxidative stresses.

We first set out to compare the translational responses of wild-type (WT) C. neoformans to temperature and oxidative stress by rapidly shifting the temperature of liquid cultures from 30°C to 37°C and by treating them with H_2_O_2_, respectively. To isolate the effects of environmental stressors, we included samples treated with 40 mM 3-amino-1,2,4-triazole (3-AT), a well-characterized inhibitor of translation. 3-AT causes ribosomes to pause at histidine codons as a result of reduced histidine biosynthesis, thereby reducing hystidyl-tRNA levels in the cell (25). The combination of these events results in Gcn2-mediated phosphorylation of eIF2α.

Western blotting revealed a modest and transient phosphorylation of eIF2α in response to the shift to 37°C (Fig. 1B). The phosphorylation was more pronounced in response to 1 mM H_2_O_2_, but 2 mM H_2_O_2_ induced a strong response that was sustained for at least 60 min. Sustained phosphorylation was also achieved with 40 mM 3-AT, although the levels of phospho-eIF2α were more similar to those observed with 1 mM H_2_O_2_.

We previously showed that C. neoformans reduces levels of ribosomal protein transcripts in response to various stressors and that the reduction in response to 37°C stress is necessary for stress-responsive translatome reprogramming (5, 43–45). Therefore, we measured the levels of RP transcript *RPL2* in response to temperature, oxidative stress, and 3-AT treatment. As expected, 37°C stress rapidly reduced *RPL2* levels (Fig. 1C). Oxidative stress also reduced *RPL2* levels, with a greater effect from 2 mM H_2_O_2_ than from 1 mM H_2_O_2_. A mild reduction in *RPL2* levels were observed in the 3-AT-treated samples, suggesting that mechanisms other than Gcn2 activation contribute to the repression seen after temperature or oxidative stress.

We performed polysome profiling to compare the overall translational states in C. neoformans exposed to temperature and oxidative stresses. The stress conditions of 37°C and 1 mM H_2_O_2_ resulted in a moderate collapse of the polysomes, similar in magnitude to the collapse seen with 3-AT treatment (Fig. 1D). Specifically, there was an increase in the 60S peak, with only a minor decrease in the 80S peak, indicative of repressed translation initiation. Under stress from 2 mM H_2_O_2_, there was a greater magnitude of polysome collapse, with a greater increase in the 60S peak, which eclipsed the 80S peak. This suggests a much greater level of translational repression than that observed in response to the other three tested conditions.

To determine whether these stressors would also induce ribosome collision, we performed disome profiling, in which disomes are visualized as a peak resistant to collapse following RNase I digestion. Disome profiles were captured after 30 min of stress treatment, corresponding to the time when we saw the greatest levels of eIF2α phosphorylation from temperature and 1 mM H_2_O_2_ stress. Surprisingly, an increase in disomes and trisomes was observed in response to 37°C stress, the stress condition which exhibited the smallest increase in eIF2α phosphorylation levels (Fig. 1E). There was no change in disome accumulation as a result of 1 mM H_2_O_2_ or 3-AT treatment, and there was a decrease in disome quantity in the 2 mM H_2_O_2_-treated samples.

Together, these data suggest that although both temperature and oxidative stress result in translational repression (as observed by eIF2α phosphorylation, reduced *RPL2* levels, and polysome collapse), the mechanisms and degrees of translational repression vary with the type and magnitude of the stress. The increase in eIF2α phosphorylation in the absence of an accumulation in ribosome collisions in peroxide is consistent with a model in which Gcn2 activation serves as a negative feedback mechanism to limit the number of ribosomes on mRNAs as the cells respond to stress. Additionally, 1 mM H_2_O_2_ stress may result in individually stalled ribosomes which are unable to collide and thus cannot be detected using an RNase I protection assay. The lack of Gcn2 activation in temperature stress compared to oxidative stress, despite an increase in ribosome collisions, suggests that the collisions occurring in temperature stress may differ than those in other stressors.

### Gcn2 is required for translational repression during the Dgcn2 oxidative stress but is dispensable during temperature stress.

**FIG 2:**
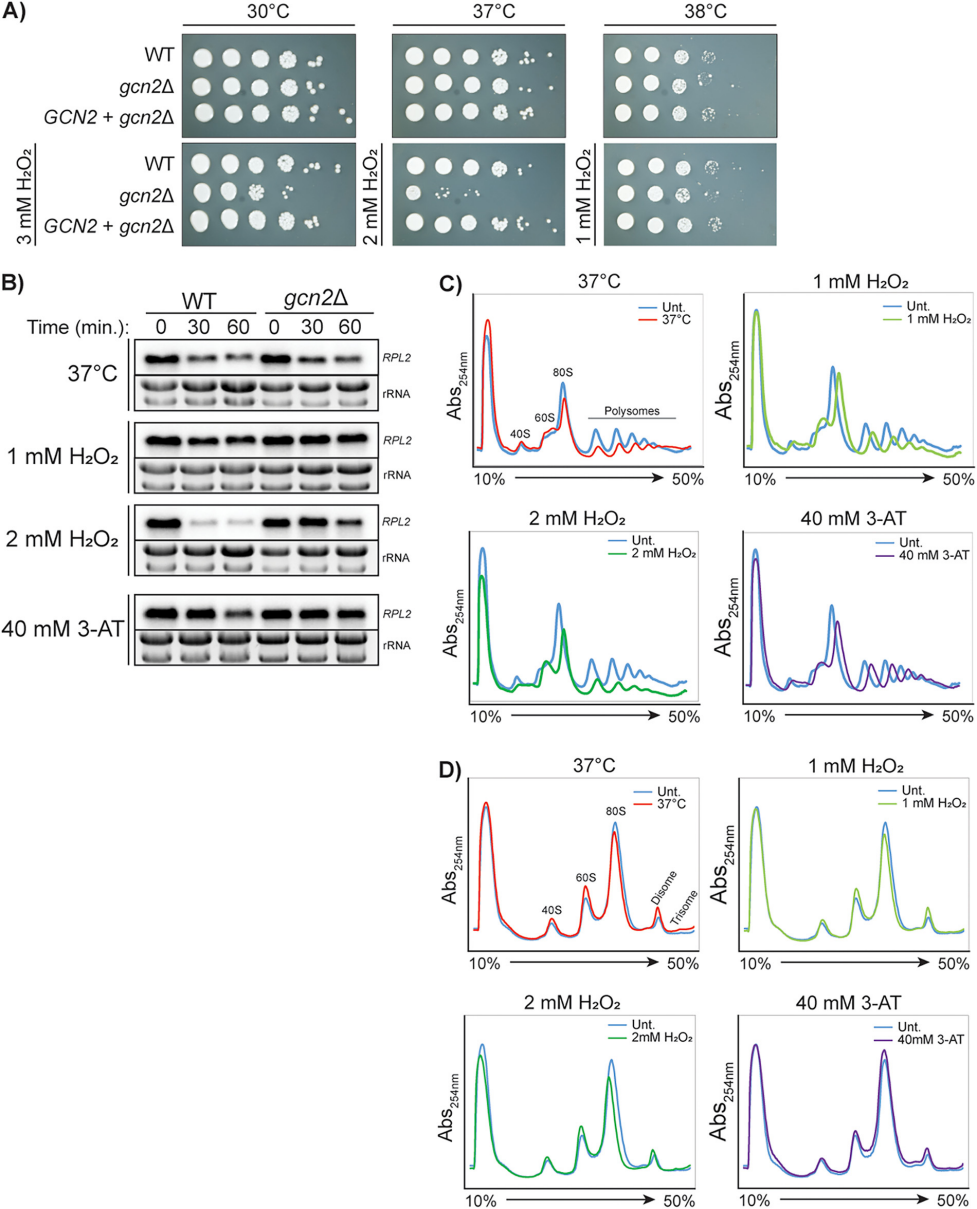
Gcn2 is primarily required for translational repression under oxidative stress. (A) Serial dilution spot plate analysis of wild-type (WT), *gcn2*Δ mutant, and *GCN2 *+ *gcn2*Δ complemented strains. (B) Northern blot analysis for *rpl2* transcripts in WT and *gcn2*Δ mutant strains in response to 37°C, 1 mM H_2_O_2_, 2 mM H_2_O_2_, and 40 mM 3-AT. (C) Polysome profiling of the *gcn2*Δ mutant in response to 30 min at 37°C, 1 mM H_2_O_2_, 2 mM H_2_O_2_, and 40 mM 3-AT. (D) Polysome profiling analysis of RNase I-digested samples from panel C.

To determine the contribution of Gcn2 to the stress adaptations we observed in C. neoformans, we compared the responses to both temperature and oxidative stresses to those in a *gcn2*Δ mutant strain that is unable to phosphorylate eIF2α. We performed serial dilution spot assays, subjecting the strains to the temperature stress, oxidative stress, and combinations of temperature and oxidative stress, which more closely mimic the conditions that occur during the course of an infection. Although we expected the *gcn2*Δ mutant to exhibit temperature sensitivity given the observed eIF2α phosphorylation and increased 60S peak in the WT strain during temperature stress (Fig. 1B and D), the growth of the *gcn2*Δ the Dgcn2 mutant did not differ from that of the WT at 37°C or 38°C (Fig. 2A). The *gcn2*Δ strain showed sensitivity to 3 mM H_2_O_2_ at 30°C. Interestingly, the growth of the *gcn2*Δ strain was severely stunted when oxidative stress and temperature stress were combined. These growth phenotypes were restored when the *gcn2*Δ mutant was complemented with the WT *GCN2* gene (Fig. 2A). These data suggest that the two stressors are not only sensed differently, but they are also additive, and that Gcn2 activity becomes more important under cumulative stress.

We previously searched for C. neoformans homologs of eIF2α kinases through BLASTp analysis and found only one protein, homologous to S. cerevisiae Gcn2 (5). To ensure that there was no residual kinase activity or other kinases phosphorylating eIF2α in the *gcn2*Δ mutant, we performed a western blot analysis of eIF2α phosphorylation. Under all conditions tested, no eIF2α phosphorylation was observed (Fig. S1B in the supplemental material).

10.1128/mbio.00196-23.1 FIG S1 (A) Western blots for eIF2α phosphorylation (top) and Gcn4 (middle) in the wild-type (WT) and *gcn2*Δ strains in response to 37°C, 1 mM H_2_O_2_, 2 mM H_2_O_2_, and 40 mM 3-AT. Arrow indicates band for Gcn4. (B) Western blots for eIF2α phosphorylation in the WT, *gcn1*Δ, *gcn2*Δ, and *gcn20*Δ strains in response to 1 mM H_2_O_2_. (C) Western blots for Gcn4 in response to 1 mM H_2_O_2_ in the WT and *gcn4*Δ strains. Recombinant Gcn4 protein was included for antibody validation. (D) Northern blot analysis for the *ARG1* and *RPL2* transcripts in response to 1 mM H_2_O_2_ and 40 mM 3-AT in the WT and *gcn4*Δ strains. Download FIG S1, TIF file, 9.7 MB. Copyright © 2023 Knowles et al. 2023 Knowles et al. https://creativecommons.org/licenses/by/4.0/ This content is distributed under the terms of the Creative Commons Attribution 4.0 International license.

Northern blots showed that *RPL2* levels were suppressed similarly in the WT and *gcn2*Δ strains under 37°C stress (Fig. 2B). However, the *gcn2*Δ mutant failed to reduce *RPL2* levels under oxidative stress with 1 and 2 mM H_2_O_2_, unlike the WT strain. Similar results were obtained with 3-AT treatment; whereas the WT showed a moderate reduction in *RPL2* at 60 min, no reduction was detectable in the *gcn2*Δ mutant.

Polysome profiling confirmed that the *gcn2*Δ mutant is defective in polysome repression in response to oxidative stress, congruent with previously reported data (5). In contrast to that for the WT strain (Fig. 1D), an increase in the 60S peak and the collapse of polysome peaks were not observed for the mutant in response to 1 mM or 2 mM H_2_O_2_ (Fig. 2C). 3-AT treatment similarly had no effect on the ribosome profile of the *gcn2*Δ mutant.

Because Gcn2 activity was recently linked to ribosome collision and subsequent quality control events (28), we hypothesized that ribosome collisions would accumulate in a strain which might be defective in recognizing stalled/collided ribosomes or transducing subsequent signaling events. Disome profiling in the *gcn2*Δ mutant after a 30-min treatment demonstrated only a modest increase in disomes, which was observed under all stress and treatment conditions (Fig. 2D). Importantly, there was a retention of disomes when the *gcn2*Δ strain was treated with 2 mM H_2_O_2_, unlike in the WT strain, which may be due to failure to effectively repress entry of oxidatively damaged mRNAs into the translating pool.

Additional components of the general control nonderepressible (Gcn) pathway have been implicated in the activation of Gcn2. Gcn1 and Gcn20 proteins are conserved in all eukaryotes. Gcn1 binds collided ribosomes and is required for full activation of Gcn2 (46–48). More recently, the structure of Gcn1 bound to stalled and collided ribosomes was resolved, further implicating ribosome collision as being a trigger for the induction of the ISR (49). We assessed single-deletion mutants of the C. neoformans orthologues *GCN1* (CNAG_03748) and *GCN20* (CNAG_04613), identified by BLASTp of the S. cerevisiae protein sequences into H99, for conserved function. We found that Gcn1 is required for full kinase activity of Gcn2 in response to oxidative stress (Fig. S1A). Gcn20, however, was found to be nonessential for Gcn2 activation (Fig. S1A).

These data indicate that Gcn2 is required for *RPL2* repression and polysome collapse during oxidative stress but is dispensable during temperature stress. However, Gcn2 improves survival under temperature stress when other stressors are present. Additionally, Gcn2-dependent regulation of translation initiation contributes to the prevention and/or resolution of the ribosome collisions that occur in response to severe oxidative stress.

### Ccr4 is required for the translational response to temperature and oxidative stresses.

**FIG 3:**
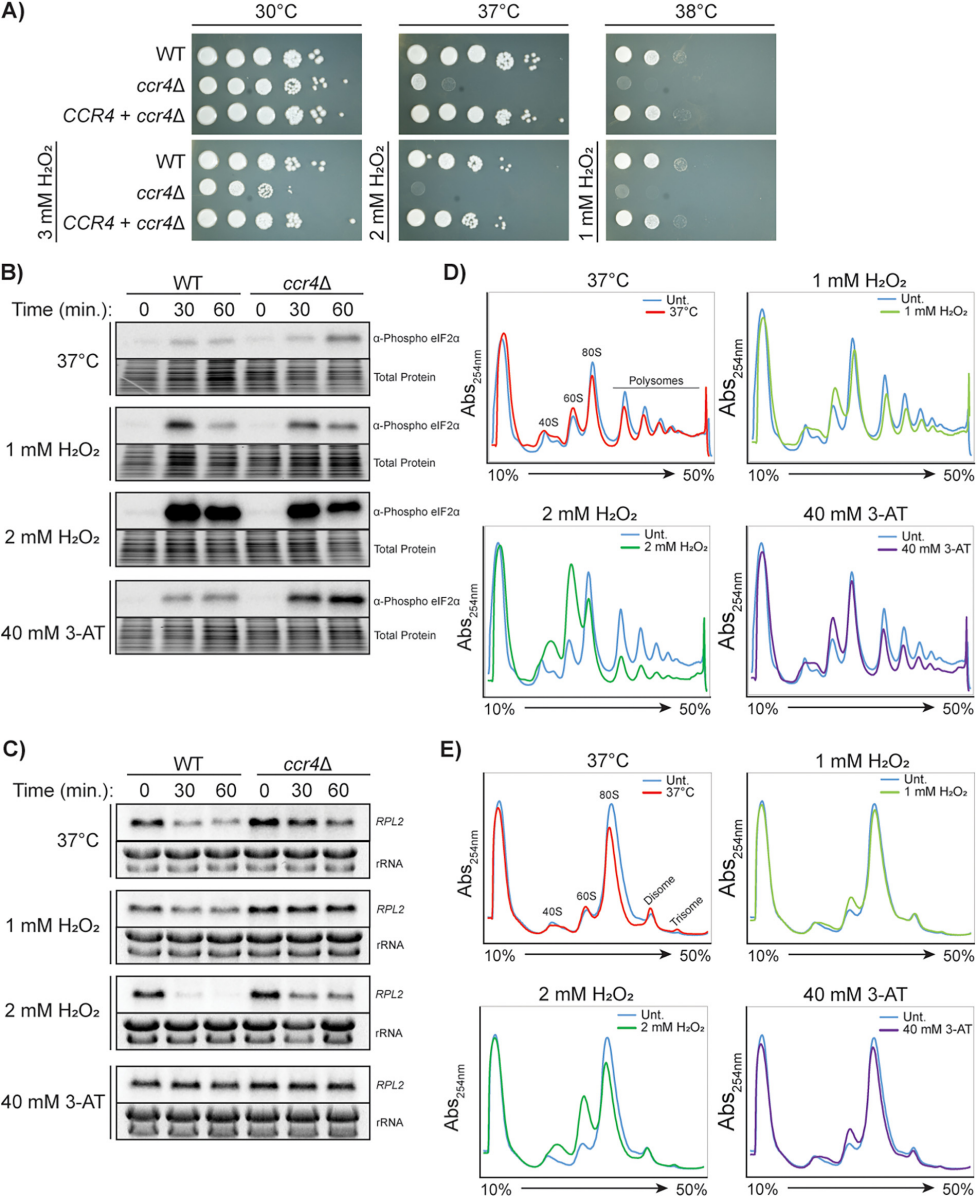
Ccr4 is required for translational repression in response to temperature and oxidative stresses. (A) Serial dilution spot plate analysis of WT, *ccr4*Δ mutant, and *CCR4* + *ccr4*Δ complemented strains. Western blots for phosphorylated eIF2α (B) and northern blots for the *RPL2* transcript (C) in WT and *ccr4*Δ strains in response to 37°C, 1 mM H_2_O_2_, 2 mM H_2_O_2_, and 40 mM 3-AT. (D) Polysome profiling of the *ccr4*Δ mutant after 30 min at 37°C, 1 mM H_2_O_2_, 2 mM H_2_O_2_, and 40 mM 3-AT. (E) Polysome profiling analysis of RNase I-digested samples from panel D.

A *ccr4*Δ C. neoformans mutant deficient in deadenlylation-dependent mRNA decay has severely attenuated virulence in a mouse model and is broadly stress-sensitive, suggesting that Ccr4 contributes to stress adaptation in general (10). We previously showed that the *ccr4*Δ mutant is defective in translatome reprogramming in response to temperature stress (2, 4). Therefore, we assessed whether this defect occurs in response to other physiologically relevant stressors, namely, oxidative stress alone and in combination with temperature stress. The results from a spot plate assay demonstrated that, in addition to its temperature sensitivity, the growth of the *ccr4*Δ mutant was also sensitive to oxidative stress at 30°C (Fig. 3A). Moreover, this defect was more pronounced under compound stress conditions of 37°C with 2 mM H_2_O_2_ and 38°C with 1 mM H_2_O_2_. All growth phenotypes were restored when the *ccr4*Δ mutant was complemented with the WT *CCR4* gene (Fig. 3A).

We then asked if translation initiation was repressed in *ccr4*Δ to compensate for the retention of mRNAs in the translating pool (3). A western blot analysis showed that while eIF2α phosphorylation began to resolve after 30 min in the wild-type sample in response to 37°C temperature stress (Fig. 1B and 3A), this response was exacerbated and prolonged in a *ccr4*Δ mutant relative to the WT and was increased overall in response to 3-AT treatment (Fig. 3B). Under both oxidative stress conditions, eIF2α phosphorylation was unchanged in the *ccr4*Δ mutant relative to the WT (Fig. 3B). Loss of Ccr4 also led to relative protection of *RPL2* transcripts in response to oxidative stresses (Fig. 3C), expanding the scope of the known role for Ccr4 in temperature stress, and suggesting that Ccr4 universally promotes RP mRNA decay in response to translational perturbations in C. neoformans.

Polysome profiling showed that, unlike the WT strain (Fig. 1D), the *ccr4*Δ strain retained low-molecular-weight polysomes and failed to accumulate high-molecular-weight polysomes under 37°C stress (Fig. 3D), consistent with its previously reported translatome reprogramming defect (3). No increase in the 60S peak was observed, also consistent with previous reports (3). Furthermore, the *ccr4*Δ strain failed to show the polysome collapse or increases in monosome and 60S peaks in response to 1 mM H_2_O_2_ that were noted in the WT strain (compare Fig. 3D and Fig. 1D). In response to 2 mM H_2_O_2_, the *ccr4*Δ mutant exhibited polysome and 80S peak collapse with an increase in subunit peaks, but not to the extent seen in the WT strain. The polysome collapse in the *ccr4*Δ mutant in response to 3-AT treatment was similar to that observed in the WT strain; however, the 60S peak did not increase to the same extent.

Surprisingly, disomes accumulated in the *ccr4*Δ mutant to a similar extent as in the WT strain under untreated, temperature, and 1 mM H_2_O_2_ conditions (Fig. 3E), suggesting that Ccr4 is not involved in clearing collided ribosomes. Unlike that in the WT strain, the disome peak was retained in the *ccr4*Δ strain after treatment with 2 mM H_2_O_2_. This suggests that the cell’s response to 2 mM H_2_O_2_ is distinct from the responses to the other three stressors tested, and that a defect in translational repression may result in perpetuation of ribosome collision.

We conclude from these data that the *ccr4*Δ mutant is defective in eliminating abundant RP transcripts (e.g., *RPL2*) under temperature and oxidative stresses, resulting in an increased sensitivity to these stressors and a reduction in fitness. Thus, deadenylation-dependent mRNA decay impacts translatome reprogramming in response to both temperature and oxidative stress, whereas regulation of translation initiation thus far appears only to impact the translational response to oxidative stress.

### Additional stress from growth in minimal medium contributes to the translational response.

To more closely represent the environment experienced by C. neoformans inside the human lung, we additionally assessed the translational response in minimal media. So far, experiments assessing translatome reprogramming have been performed in nutrient-rich yeast extract-peptone-dextrose (YPD) medium. During pathogenesis, C. neoformans is not only exposed to temperature and oxidative stress simultaneously, but also to nutrient limitation. We reasoned that the additional stress of growth in a minimal medium (with only dextrose and ammonium as sources of carbon and nitrogen, respectively), would further impact translational capacity. Thus, we compared C. neoformans strains grown in YPD to those grown in yeast nitrogen base (YNB) with dextrose. To model this response, we created a compound stress environment by treating cells with varying combinations of temperature (30°C or 37°C), oxidative stress (0 mM, 0.5 mM, and 1.0 mM H_2_O_2_), and nutrient availability (YPD or YNB + 2% dextrose).

**FIG 4:**
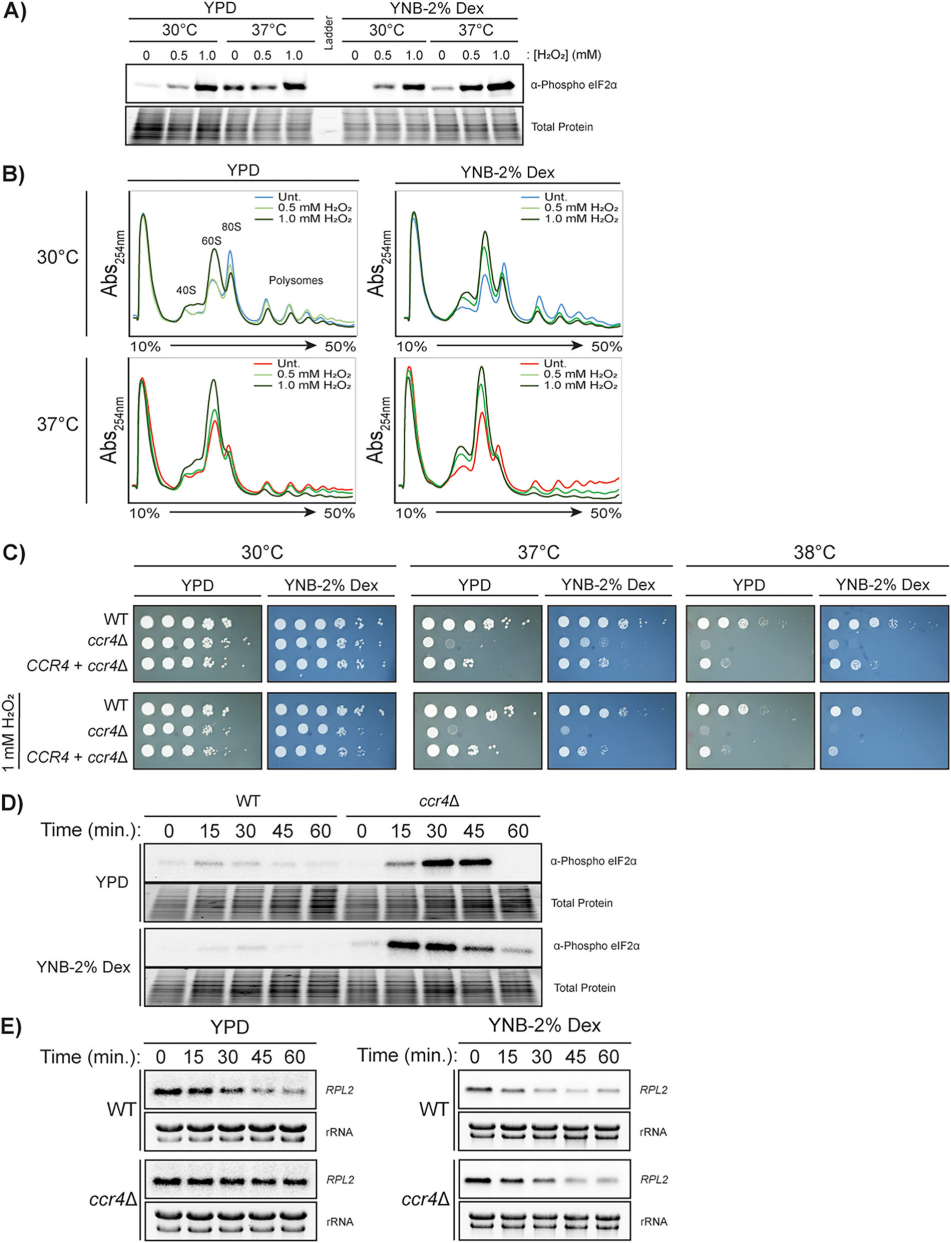
Minimal medium is a stressful environment that results in increased repression of translation initiation. (A) Western blots for phosphorylated eIF2α in a WT strain after 30 min of treatment in response to combinations of 30°C and 37°C temperature stress, 0 mM H_2_O_2_, 0.5 mM H_2_O_2_, or 1 mM H_2_O_2_ stress, in either yeast extract-peptone-dextrose (YPD) (complete) or yeast nitrogen base (YNB) + 2% dextrose (YNB-2% Dex) (minimal) medium. (B) Polysome profiling of the conditions in panel A. (C) Serial dilution spot plate analysis of WT, *ccr4*Δ mutant, and *CCR4 *+ *ccr4*Δ complemented strains. (D) Western blots for phosphorylated eIF2α in WT and *ccr4*Δ mutant strains in response to 37°C in nutrient-rich medium (YPD) and minimal medium (YNB-2% Dex). (E) Northern blot analysis for *RPL2* transcripts in WT and *ccr4*Δ mutant strains in response to 37°C in either YPD or YNB-2% dextrose.

We assessed eIF2α phosphorylation after 30 min of treatment with all possible compound stress conditions. As expected, we saw a dose-dependent increase in eIF2α phosphorylation with increasing H_2_O_2_ stress (Fig. 4A). The addition of temperature stress only had a modest impact on overall eIF2α phosphorylation, and minimal media did not exhibit an appreciable change. When assessed by polysome profiling, the profiles from cultures grown in YPD and minimal medium in the absence of additional stress appeared similar. The intermediate H_2_O_2_ concentration of 0.5 mM at 30°C in YPD caused no increase in 60S peak height, whereas there was a substantial increase in 60S peak height at the same concentration in minimal medium with a concomitant collapse in polysomes. At 37°C in the absence of H_2_O_2_, these two media again resulted in similar profiles, with increased 60S peak height and a loss of polysomes relative to 30°C. However, the intermediate 0.5 mM H_2_O_2_ concentration led to higher 60S peak height in minimal medium. This suggests that cells grown in minimal media exhibit a similar translational response to temperature stress but exhibit a lower threshold for increased repression of translation initiation.

When assessing the *ccr4*Δ strain by spot plate analysis, the *ccr4*Δ mutant grew better on YNB than on YPD at 37°C (Fig. 4C). This was also observed in the WT strain at 38°C, although to a lesser extent (Fig. 4C). However, the protection afforded by minimal medium (i.e., YNB) was lost under conditions of oxidative stress, i.e., when 1 mM H_2_O_2_ was added. These data suggest that growth on minimal media allows for a limited capacity of stress priming, affording the *ccr4*Δ mutant a level of protection.

To further investigate this potential for stress priming, we assessed eIF2α phosphorylation in the *ccr4*Δ mutant in complete and minimal media, in response to temperature stress. The phosphorylation of eIF2α in response to 37°C stress was initiated sooner in the *ccr4*Δ strain grown in minimal medium (Fig. 4D). We then investigated whether this enhancement affected the clearance of *RPL2* from the translating pool. Indeed, *RPL2* levels were reduced in the *ccr4*Δ strain when it was grown in YNB medium, resulting in a reduction in *RPL2* transcripts which paralleled that in the WT (Fig. 4E).

These data suggest that increased eIF2α phosphorylation levels, or otherwise altered translation kinetics in minimal media, contribute to translatome reprogramming in the absence of Ccr4-mediated clearance of RP transcripts, conferring some degree of protection. The difference we observed between minimal media and rich media highlight the impact of metabolic need on the translational response of C. neoformans to stress.

### Gcn2 activation suppresses the defects in RP repression and polysome collapse of *ccr4*Δ in minimal medium.

**FIG 5:**
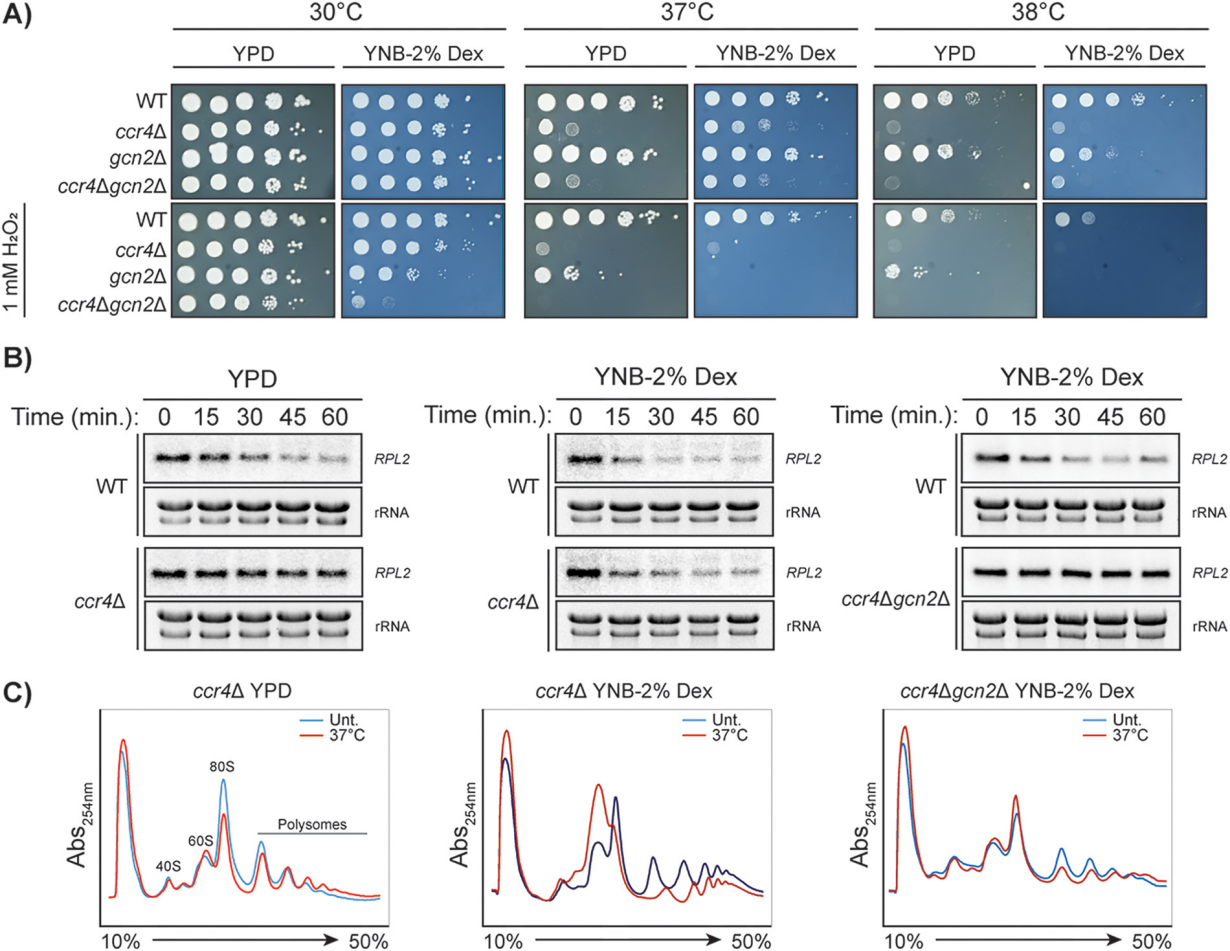
Gcn2-mediated translational repression in minimal medium can suppress the mRNA decay defect of *ccr4*Δ during temperature-responsive translatome reprogramming. (A) Serial dilution spot plate analysis of WT, *ccr4*Δ, *gcn2*Δ, and *ccr4*Δ*gcn2*Δ mutant strains at different temperatures and in the presence of 1 mM H_2_O_2_ in YPD or YNB-2% dextrose. (B) Northern blot analysis for *RPL2* transcripts in WT and *ccr4*Δ or *ccr4*Δ*gcn2*Δ strains in response to 37°C in either YPD or YNB-2% dextrose. (C) Polysome profiling of *ccr4*Δ and *ccr4*Δ*gcn2*Δ mutants in either YPD or YNB-2% dextrose after 30 min at 37°C.

To determine whether eIF2α phosphorylation is a contributing factor to the protection of the *ccr4*Δ mutant in minimal media, we generated a *ccr4*Δ*gcn2*Δ double mutant. Growth on YNB medium afforded the *ccr4*Δ*gcn2*Δ mutant some protection against 37°C stress, similar to that observed for the *ccr4*Δ mutant, but not against oxidative stress (Fig. 5A). The combinatorial stress of 37°C and 1 mM H_2_O_2_ synergistically affected growth of the *ccr4*Δ*gcn2*Δ mutant, regardless of the medium, which is in agreement with the data showing that both Ccr4 (Fig. 3A) and Gcn2 (Fig. 2A) contribute to fitness under oxidative stress. However, the *ccr4*Δ*gcn2*Δ double mutant did not differ from the *ccr4*Δ mutant when under temperature stress alone on either medium, also consistent with the data showing that Ccr4, but not Gcn2, contributes to the temperature stress response (Fig. 2A).

The reduction in *RPL2* at 37°C that was observed in the *ccr4*Δ mutant grown on minimal medium (Fig. 5B, center; Fig. 4E) was abolished in the *ccr4*Δ*gcn2*Δ mutant (Fig. 5B right), suggesting that Gcn2 mediates the repression of RP mRNA in C. neoformans grown in minimal medium. Accordingly, there was no evidence of polysome collapse in the *ccr4*Δ*gcn2*Δ double mutant grown in YNB minimal medium at 37°C (Fig. 5C, right), which was observed as a larger 60S peak and concomitant smaller 80S peak in the *ccr4*Δ mutant in response to 37°C in the same medium (Fig. 5C, center). This suggests that although Gcn2 is dispensable for temperature adaptation in YPD, Gcn2-dependent eIF2α phosphorylation drives the translational response of the *ccr4*Δ mutant to temperature stress in minimal medium. This may indicate that growth in minimal medium is perceived as a translational stressor which, when compounded with temperature, leads to Gcn2 activation. These data also suggest that Gcn2 activation can compensate for the loss of Ccr4 in minimal medium with regard to translational repression (i.e., ribosome collapse and RP clearance), but this is not sufficient to maintain growth.

### Translational stress induces the ISR.

In addition to repressing cap-dependent translation initiation, Gcn2-mediated eIF2α phosphorylation in yeast simultaneously promotes the translation of the transcription factor Gcn4, inducing the ISR (13). ISR regulons, defined as the transcriptional targets of Gcn4, have been characterized in S. cerevisiae, and in filamentous ascomycetes, as the cross-pathway control (*cpcA*, *cpc-1*) regulon (50). In S. cerevisiae, ISR induction is a pro-growth, stress-responsive reprogramming event which results in the transcription of factors involved in amino acid metabolism and oxidative stress resistance, and contributes to the stress-responsive repression of RP transcripts (18, 51). The ISR in C. neoformans remains uninvestigated at this time, and the regulation of ISR-induced stress mitigation is greatly important to understanding the ability of C. neoformans to adapt to the host environment.

**FIG 6:**
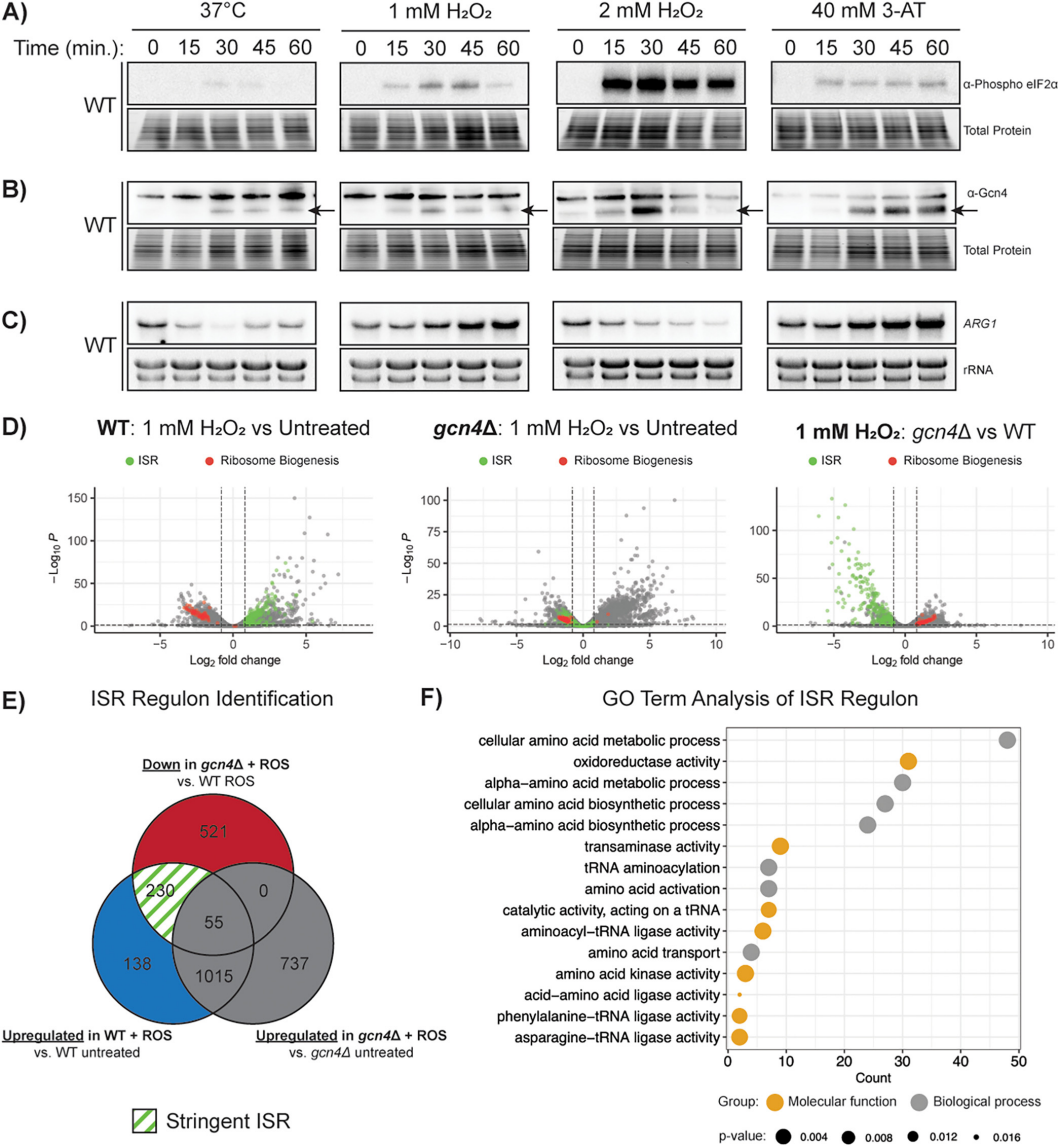
Temperature and oxidative stresses result in differential integrated stress response (ISR) induction. (A) Western blots for eIF2(alpha) phosphorylation in the WT strain in response to 37°C, 1 mM H_2_O_2_, 2 mM H_2_O_2_, and 40 mM 3-AT. (B) Western blots for Gcn4 under the same conditions as panel A. Arrows indicate the band for Gcn4 (C) Northern blot analysis for the *ARG1* transcript under the same conditions as for panel A and B. (D) Volcano plots of –log_10_ *P* values versus log_2_ fold-change for RNA levels in the WT strain (1 mM H_2_O_2_-treated versus untreated; left), *gcn4*Δ mutant (1 mM H_2_O_2_-treated versus untreated; center), and the *gcn4*Δ mutant and WT (1 mM H_2_O_2_; right). Genes identified as part of the ISR are shown in green and ribosome biogenesis genes are shown in red. (D) Venn diagram describing the rationale used to determine the stringent ISR regulon. (F) Gene Ontology (GO) enrichment analysis of genes identified as components of the ISR regulon.

To determine which stresses trigger the ISR in C. neoformans, we measured the protein levels of Gcn4, the expression of which is canonically upregulated when eIF2α is phosphorylated (15). We expected to see increased expression under conditions that induced eIF2α phosphorylation (Fig. 6A and Fig. 1B), and thus the greatest induction in response to 2 mM H_2_O_2_ (Fig. 6A and Fig. 1B). However, Western blotting showed only a moderate increase in Gcn4 protein in response to 2 mM H_2_O_2_ (Fig. 6B, arrow indicates the band for Gcn4). Furthermore, 37°C stress and 1 mM H_2_O_2_ similarly induced Gcn4 despite greater phosphorylation of eIF2α in response to the oxidative stress (Fig. 6A and Fig. 1B). Also, 1 mM H_2_O_2_ and 40 mM 3-AT induced similar levels of eIF2α phosphorylation (Fig. 6A and Fig. 1B), but 40 mM 3-AT resulted in a much greater increase in Gcn4 protein levels (Fig. 6B). These observations indicate that only modest levels of eIF2α phosphorylation are necessary to trigger uORF bypass and subsequent translation of the Gcn4 protein-coding ORF, and that the amount of translated Gcn4 does not scale with the magnitude of eIF2α phosphorylation.

To further assess ISR induction, we examined the expression pattern of *ARG1*, a known target of Gcn4 in Saccharomyces cerevisiae (16). After confirming that *ARG1* is a bona fide target of Gcn4 in C. neoformans using the *gcn4*Δ mutant (Fig. S1D), we measured *ARG1* expression during 60 min of exposure to each stress. Northern blotting revealed that 1 mM H_2_O_2_ and 40 mM 3-AT induced *ARG1* expression, 2 mM H_2_O_2_ suppressed its expression, and 37°C suppressed its expression only transiently (Fig. 6C).

Because the ISR is currently undescribed in C. neoformans, we performed RNA sequencing to identify the transcriptome in response to 60 min of oxidative stress (1 mM H_2_O_2_) in the wild type and *gcn4*Δ. We initially defined the ISR regulon as transcripts that were downregulated (fold change [FC] < 1.75, *P* < 0.05) in the *gcn4*Δ mutant compared to WT in the context of oxidative stress (806 transcripts) (Fig. 6E). However, this returned a substantial number of transcripts which either were not upregulated by the oxidative stress response or were upregulated even in the absence of Gcn4. We employed a more stringent analysis by focusing only on the transcripts which experienced Gcn4-dependent upregulation in the WT strain in response to oxidative stress (230 transcripts), which we defined as the C. neoformans ISR regulon (Fig. 6E, Table S2). A Gene Ontology (GO) term analysis was then performed on the 230 transcripts comprising the ISR regulon, revealing that the ISR is primarily comprised of components of amino acid biosynthetic pathways and tRNA regulation, and, critically for the response to oxidative stress, genes with oxidoreductase activity (Fig. 6F).

10.1128/mbio.00196-23.3 TABLE S2 Differentially regulated genes from RNA sequencing of WT and *gcn4*Δ after 1 h of treatment with 1mM H_2_O_2_. Download Table S2, XLSX file, 0.5 MB. Copyright © 2023 Knowles et al. 2023 Knowles et al. https://creativecommons.org/licenses/by/4.0/ This content is distributed under the terms of the Creative Commons Attribution 4.0 International license.

Taken together, our observations demonstrate that the ISR in C. neoformans is induced by various host-relevant stresses when assessed by Gcn4 translation; however, the induction of one of its downstream targets, *ARG1*, varies according to the degree and type of stress. We also defined the C. neoformans ISR regulon, which closely matches that seen in the S. cerevisiae literature with respect to the regulation of amino acid and tRNA metabolism (16).

## DISCUSSION

Part of the success of C. neoformans as a pathogen is its ability to respond and adapt to the environment inside the human lung. Although it is known that this requires mRNA decay-dependent translatome reprogramming (3, 4), the mechanisms regulating this are unclear. We provide evidence supporting the roles of Ccr4-dependent mRNA decay and Gcn2-dependent eIF2α phosphorylation.

Our data demonstrate that temperature stress and various degrees of oxidative stress are sensed differently by C. neoformans and result in different outcomes. Temperature stress resulted in rapid clearance of RP mRNAs and modest eIF2α phosphorylation, corresponding to a distinctly observable increase in collided ribosomes. Mild oxidative stress (from 1 mM H_2_O_2_) resulted in minimal RP mRNA repression and notable eIF2α phosphorylation with no increase in ribosome collisions, despite previous literature tying Gcn2 activation to ribosome collisions (28, 31, 49). Interestingly, temperature stress and mild oxidative stress produced similar polysome profiles. These data, when viewed in the context of recent literature (24, 52), suggest that temperature stress and mild oxidative stress result in differing states of stalled or collided ribosomes. Recent studies have revealed that differing occupancy of tRNA binding sites on the ribosome results in the activation of different downstream quality control pathways (24, 52); specifically, an empty A site is required for Gcn2 activation (24). This would suggest that the collided ribosomes as a result of temperature stress are stalled with an occupied A site rather than the empty A site required for Gcn2 activation. Disome accumulation in response to temperature stress has not been observed in S. cerevisiae. Therefore, further investigation into this sensing mechanism may illuminate specific pathways relevant to thermotolerance in fungal pathogens.

We observed an association between ribosome collisions and accelerated RP mRNA repression. Recently, a role for Not5 in the link between ribosome collisions and mRNA surveillance and decay pathways was described, where Not5 interacts with a transiently unoccupied ribosome E site and recruits the Ccr4-NOT complex (52). Our data demonstrate that the translational response to temperature is driven mainly by Ccr4-dependent mRNA decay, whereas the response to oxidative stress relies more heavily on Gcn2 activation. Thus, temperature stress may lead to ribosome collisions, resulting in ribosomes with unoccupied E sites and accelerated Ccr4-mediated mRNA decay, whereas mild oxidative stress results in stalled ribosomes with unoccupied A sites, leading to increased Gcn2 activation.

Severe oxidative stress (from 2 mM H_2_O_2_) promoted RP mRNA decay and translational repression by a mechanism different from that induced by temperature and mild oxidative stress, possibly one that is not coordinated by the cell and is instead a result of cellular damage. Oxidation of mRNAs can lead to decoding errors, and oxidation of ribosomal proteins and rRNA may result in dysfunctional ribosomes (53, 54). Our observed lack of induction of *ARG1* in response to 2 mM H_2_O_2_ may even suggest that sufficient DNA damage precluded the transcriptional activation and translation of Gcn4. Nevertheless, C. neoformans can survive this stress, with no loss of viability on spot plates. This suggests that as a highly adaptive human pathogen, C. neoformans has multiple pathways to sense, respond to, and detoxify reactive oxygen species.

Growth in minimal medium exacerbates oxidative stress phenotypes while mitigating the responses to temperature stress. The mechanism responsible for this involves Gcn2 because the loss of *GCN2* in the double mutant reversed the loss of translational repression observed in the *ccr4*Δ mutant at 37°C in minimal medium. Thus, we propose an eIF2α phosphorylation “stress threshold” hypothesis in which cells grown in minimal medium are closer to the threshold of Gcn2 activation. These cells are then easily pushed over the threshold by temperature stress, which triggers the protection afforded by translational repression and ISR induction. Because of the stress from obligate anabolism in minimal medium, the cells express mRNAs encoding enzymes required for anabolism, which can be longer than RP mRNAs and may more readily trigger translational pausing.

The recent identification of Gcn4 in C. neoformans enabled us to begin studying the role and regulation of the ISR (15). Gcn2 activity was required for the translation of Gcn4, providing the first evidence, to our knowledge, of this conserved translational regulatory mechanism in basidiomycete fungi. Gcn4 translation did not necessarily correspond to eIF2α phosphorylation, although the modest levels of phospho-eIF2α induced by temperature and mild oxidative (1 mM H_2_O_2_) stresses were sufficient. This is consistent with the model in which phosphorylated eIF2α limits the availability of the 43S pre-initiation complex and promotes the bypass of the upstream open reading frame of the mRNA for Gcn4 (15). Under severe oxidative stress (from 2 mM H_2_O_2_), when the levels of Gcn4 and eIF2α phosphorylation were highest, the transcription of ISR effector *ARG1* was repressed rather than induced. This suggests that there is a maximum level at which cells can cope with stress through the ISR, likely as a result of reduced transcription following this level of oxidative insult. Finally, the ISR regulon comprises a part of the translatome reprogramming in which translational resources are directed to stress-responsive mRNAs.

Together, these data support a model in which C. neoformans senses stress by monitoring its translational state and reprograms its translatome to combat the encountered stress. The differential responses of C. neoformans to temperature and oxidative stresses are particularly important because this organism is an environmental fungus, and the ability to survive at higher temperatures is crucial to the pathogenicity of this and other fungi to humans and other animals. Moreover, increasing global surface temperatures will likely select for thermotolerance in other environmental pathogens. It is imperative that future work determines the molecular mechanisms that sense temperature stress and defines those responsible for promoting the stress-responsive translatome.

## MATERIALS AND METHODS

### Strains.

C. neoformans var. *grubii* H99 serotype A (taxid: 235443) was used as the WT strain in this study. All mutant strains were created in this WT background. The *ccr4*Δ strain used was previously published (2). Other mutant strains were constructed using previously described methods (55). To make the *ccr4*Δ*gcn2*Δ strain, a *gcn2*Δ strain was first constructed as described previously (5), with the exception that the homologous arms were flanked by a G418 resistance cassette amplified with the primers F-NEO-BglII and R-NEO-SacI (primers listed in Table S1) and used to transform the WT strain. The *gcn2*Δ strain was then transformed with the *CCR4* knockout construct previously published (2). The *CCR4 *+ *ccr4*Δ strain was constructed by amplifying the *CCR4* genomic locus from the WT strain using primers F-CCR4-1kbUp-SpeI and R-CCR4-1kbDown-SpeI (Table S1) and inserting it into the *ccr4*Δ mutant (2). The *gcn4*Δ strain was obtained from the Fungal Genetics Stock Center at the University of Kansas as part of the Madhani 2015 knockout collection. The *gcn1*Δ mutant was created in the H99 background by first obtaining the *gcn1*Δ mutant from the Madhani 2015 knockout collection and amplifying the *gcn1*Δ genomic locus, ±1,000 bp where *GCN1* was replaced with the nourseothricin resistance cassette using primers Gcn1-KO-FWD and Gcn1-KO-REV. The amplified *gcn1*Δ genomic locus was then introduced into the H99 background as previously described (55). The *gcn20*Δ mutant was created using the same method described above for *gcn1*Δ using primers Gcn20-KO-FWD and Gcn20-KO-REV. All strains were confirmed by PCR, Northern blotting, and phenotypic profiling.

10.1128/mbio.00196-23.2 TABLE S1 Oligonucleotide primer sequences used in this study. Download Table S1, DOCX file, 0.01 MB. Copyright © 2023 Knowles et al. 2023 Knowles et al. https://creativecommons.org/licenses/by/4.0/ This content is distributed under the terms of the Creative Commons Attribution 4.0 International license.

### Media and growth conditions.

All experimental cultures were started from overnight cultures grown in YPD (1% yeast extract, 2% peptone, and 2% dextrose) at 30°C. Experimental cultures were seeded to an optical density at 600 nm of 0.18 to 0.20 in YPD (unless otherwise noted). YNB (BD Difco cat no. 291920) supplemented with 2% dextrose was used as the minimal medium. Cultures were grown at 30°C until the mid-logarithmic phase was reached. Cells were pelleted at 3,000 rcf for 2 min and resuspended in prewarmed 37°C medium for temperature stress or with medium containing 1 mM or 2 mM H_2_O_2_ (Fisher Chemical, cat no. H325-500) for oxidative stress or 40 mM 3-AT (TCI cat no. A0432). Cultures were incubated for the times indicated in the text and figures prior to harvesting. Cells were harvested by pelleting for 2 min at 3,000 rcf and then flash-frozen in liquid nitrogen.

### Spot plating.

Strains were grown in YPD overnight at 30°C. The following day, cells were pelleted at 3,000 rcf for 2 min and washed twice in sterile deionized water. The optical density at 600 nm of each culture was set to 1.0 in sterile deionized water, and the cultures were then serially diluted 10-fold six times. Five μL was then spotted onto either YPD or YNB + 2% dextrose agar, with or without H_2_O_2_, and incubated at 30°C, 37°C, or 38°C for 72 h.

### Polysome and disome profiling.

Polysome and disome profiling was performed as previously described (56). Briefly, each cell pellet was resuspended in 1 mL of polysome lysis buffer (20 mM Tris-HCl [pH 8.0], 140 mM KCl, 5 mM MgCl_2_, 1% Triton X-100, 25 mg/mL heparin sodium sulfate, and 0.1 mg/mL cycloheximide) and transferred to a microcentrifuge tube. Cells were re-pelleted by centrifugation at 2,350 rcf for 2 min, resuspended in 50 μL polysome lysis buffer, and layered on glass beads for lysis using a Bullet Blender cooled with dry ice (5 min at setting 12). An additional 150 μL of lysis buffer was added to the glass beads. Lysate was removed from the glass beads and centrifuged at 20,050 rcf for 10 min. The cleared cell lysate was removed from the pellet, and RNA was quantified using a NanoDrop (Thermo Fisher Scientific). For each profile, 250 μg of RNA was loaded onto a 10% to 40% sucrose gradient and centrifuged at 260,800 rcf for 120 min in a SW41Ti swinging bucket rotor. A Brandel tube piercer was used to determine the RNA absorbance of the gradients at 254 nm with a Teledyne UA-6 detector. Data were captured using a DATAQ DI-1110 and recorded using DATAQ WinDaq software.

### Western blotting.

Pelleted cells were thawed and resuspended in 1 mL of sterile deionized water and transferred to 1.5-mL microcentrifuge tubes. Cells were again pelleted by centrifugation at 2,400 rcf for 2 min and resuspended in 50 μL of lysis buffer (10 mM HEPES, 100 mM KCl, 5 mM MgCl_2_, 0.5% NP-40, 10 μM dithiothreitol, and 10 μL/mL Halt protease inhibitor). Cells were layered onto glass beads and lysed using a Bullet Blender as described above. Lysate was washed from the glass beads using an additional 30 μL of lysis buffer. The whole-cell lysate was then cleared by centrifugation at 21,000 rcf for 10 min. Protein was quantified using a Pierce 660nm Protein Assay kit, and equivalent amounts of protein from each sample were boiled in Laemmli buffer containing 2-mercaptoethanol before loading onto Bio-Rad stain-free Tris-glycine gels for electrophoretic separation. Total protein was quantified by imaging the stain-free gels using a Bio-Rad Gel Doc XR+ and Image Lab software. Protein was transferred to polyvinylidene difluoride membranes using a Bio-Rad Trans-blot Turbo, and membranes were blocked with Bio-Rad EveryBlot blocking buffer. Blots were probed for phosphorylated eIF2α using anti-eIF2α (phospho-S51) antibody (Abcam, cat no. ab32157; 1:1,000 dilution). Blots were probed for Gcn4 using a polyclonal rabbit anti-Gcn4 antibody raised against recombinant C. neoformans Gcn4 by GenScript and verified for epitope specificity (Fig. S1C). Horseradish peroxidase-conjugated anti-rabbit IgG (Cell Signaling Technologies, cat no. 7074; 1:10,000 dilution) was used as the secondary antibody. Horseradish peroxidase was visualized via Bio-Rad Clarity Max (cat no. 1705062) and imaged on a Bio-Rad ChemiDoc MR. Quantification of blots was performed in Bio-Rad Image Lab.

### Northern blotting.

Cells were thawed and resuspended in Qiagen RLT buffer containing 10 μL/mL 2-mercaptoethanol. The lysates were layered in glass beads and mechanically lysed using a Bullet Blender as described above. Lysates were flushed from the glass beads using the same buffer, and whole-cell lysates were cleared by centrifugation at 21,000 rcf for 10 min. The supernatants were removed, and equal volumes of 70% ethanol were added to precipitate nucleic acids. RNA was then isolated using the Qiagen RNeasy kit according to the manufacturer’s instructions. RNA was quantified using a NanoDrop, and 5 μg was loaded onto an agarose-formaldehyde gel for electrophoretic separation. Total RNA was imaged using a Bio-Rad Gel Doc and ImageLab software. RNA was transferred to a nylon membrane and probed using a ^32^P-labeled DNA probe, created using Invitrogen RadPrime DNA Labeling kit (cat no. 18428011) as described previously (57). The *RPL2* probe was created as described previously (57). The *ARG1* probe was created using genomic DNA amplified using ARG1-Northern-F and ARG1-Northern-R primers (listed in Table S1). Blots were exposed to a phosphor screen, and the screen was scanned using a GE Typhoon phosphoimager. Signals were quantified using Quantity One software.

### RNA sequencing and analysis.

WT and *gcn4*Δ strains were grown to mid-logarithmic phase at 30°C, and cells were treated with 1 mM H_2_O_2_ or left untreated for 1 h. RNA was extracted as described for Northern blotting and treated using an on-column DNase kit (Qiagen). RNA quality was determined by investigating integrity on an RNA gel prior to sequencing. RNA samples were submitted to Genewiz (Azenta) for poly(A)+ purification, library preparation, and Illumina sequencing. The sequencing data can be viewed at GEO under accession no. GSE206508. Two biological replicates were analyzed per strain. RNA sequencing reads were trimmed to remove adapter sequences using Cutadapt (58). Filtered reads were aligned to the C. neoformans H99 genome (FungiDB) using STAR alignment (59). Read counts for each gene were calculated using RSEM (60), and differentially expressed genes across samples were determined using DESeq2 in R (61). Data were then filtered according to a 1.75-fold change and an adjusted *P* value threshold of ≤0.05.

The ISR was defined by the following three criteria: (i) genes that were downregulated in the *gcn4*Δ strain treated with 1 mM H_2_O_2_ compared to expression in the WT treated with 1 mM H_2_O_2_, (ii) genes that were upregulated in the WT upon treatment with 1 mM H_2_O_2_, and (iii) genes that were not upregulated in the *gcn4*Δ mutant upon treatment with 1 mM H_2_O_2_. Differential expression of genes was visualized using the R package EnhancedVolcano (62), with ISR genes highlighted in green and the ribosome biogenesis GO term (GO: 0042254) highlighted in red. GO analysis of the ISR genes was performed using FungiDB and visualized using the R package ggplot2 (63).

### Data availability.

The sequencing data can be viewed at GEO under accession no. GSE206508.
