# Supplementary figures and images for: Contributions of Ccr4 and Gcn2 to the Translational Response of C. neoformans to Host-Relevant Stressors and Integrated Stress Response Induction
Source: mBio. 2023 Apr 5;14(2):e00196-23. doi: 10.1128/mbio.00196-23 (PMC10127693; doi:10.1128/mbio.00196-23)

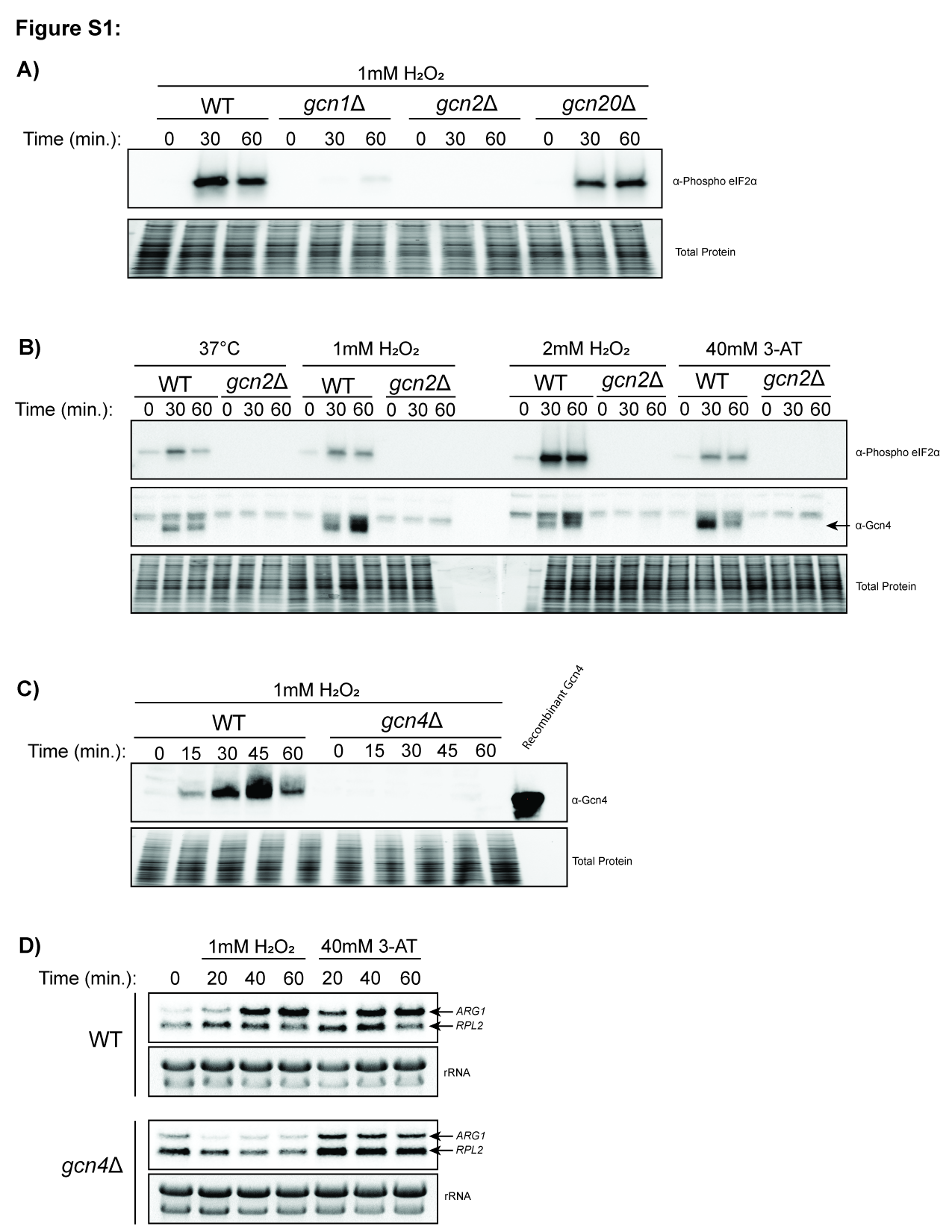

Supplement: FIG S1 [file mbio.00196-23-s0001.tif]
