# Supplementary material for: Contributions of Ccr4 and Gcn2 to the Translational Response of C. neoformans to Host-Relevant Stressors and Integrated Stress Response Induction
Source: mBio. 2023 Apr 5;14(2):e00196-23. doi: 10.1128/mbio.00196-23 (PMC10127693; doi:10.1128/mbio.00196-23)
Supplement: TABLE S1 [file mbio.00196-23-s0002.docx]

**Table S1. Oligonucleotide primers sequences**

| **Primer name** | **Sequence** |
| --- | --- |
| F-NEO-*Bgl*II | TAATAAAGATCTAGCGGATAACAATTTCACACAGG |
| R-NEO-*Sac*I | TAATAACAGCTCCGACGGCCAGTGAATTGTAATACG |
| F-CCR4-1kbUp-Spe1 | TAATAAACTAGTCTGCTGTTTCAACTCCATAGGC |
| R-CCR4-1kbDown-Spe1 | TAATAAACTAGTGGAATAGTTTGACGGGTGG |
| ARG1-Northern-F | GGTGCTGTCTTCCATCTTG |
| ARG1-Northern-R | ACAGTCTTTTGAGATGCGGGGA |
| Gcn1-KO-FWD | GTGGACGAGGTCAATAACGGGA |
| Gcn1-KO-REV | ACGTCAAGCCTTCCCTCCTATG |
| Gcn20-KO-FWD | CACCCTCGGTTCTGATCACCTT |
| Gcn20-KO-REV | ACAGACTGATGGCCCTCTCAAC |
